# Intrathoracic Goitre Extending Into the Posterior Mediastinum: A Case Report

**DOI:** 10.7759/cureus.96565

**Published:** 2025-11-11

**Authors:** Sharjeel Mahmood, Abuharaira Sabir, Talat Waseem, Hadin D Khan, Murtaza Hussain, Muqadas Riaz, Zainab Sajjad

**Affiliations:** 1 Department of General Surgery, Shalamar Hospital, Lahore, PAK; 2 Department of Gastroenterology/Transplant Hepatology, Pakistan Kidney and Liver Institute and Research Centre, Lahore, PAK; 3 Department of Community Medicine, Shalamar Medical and Dental College, Lahore, PAK

**Keywords:** compressive symptoms, median sternotomy, mediastinal mass, posterior mediastinum, retrosternal goitre, thyroid surgery

## Abstract

Intrathoracic goitre (ITG) with posterior mediastinal extension is rare and may present with compressive respiratory symptoms. We report a 55-year-old woman with long-standing shortness of breath and a history of thyroid surgery. Examination revealed only minimal cervical swelling, but imaging demonstrated a large posterior mediastinal mass consistent with an ITG. The mass was successfully excised through a combined cervical and sternotomy approach, and the patient made an uneventful recovery. This case underscores the need to consider ITG in patients with unexplained respiratory symptoms even when cervical findings are minimal, and it highlights the value of imaging and timely surgical intervention in preventing complications.

## Introduction

Since Katlic et al. first described it in 1985, several definitions of intrathoracic goitre (ITG) have been proposed, with the most widely accepted being a goitre in which at least 50% of the tissue lies below the thoracic inlet [1]. ITG may be classified as either secondary or primary (true ITG). Secondary ITG represents the downward extension of a cervical goitre into the thorax, whereas true ITG has no connection with the cervical thyroid and is located entirely within the thoracic cavity, sometimes connected by a thin, often unrecognised pedicle [2]. With the advent of standardised surgical techniques and improved access to advanced imaging, detection rates of ITG have increased in recent decades.

The majority of ITGs follow the anterior mediastinal route, corresponding to the natural descent of the thyroid gland. Posterior mediastinal extension is uncommon, accounting for only 10%-15% of ITGs in earlier reports [3]. In Pakistan, the prevalence of multinodular goitre has been reported to reach up to 30% in iodine-deficient regions; however, data on cases extending into the posterior mediastinum remain scarce [4]. Posterior ITGs are clinically significant because they may compress vital mediastinal structures, producing symptoms such as dyspnoea, stridor, dysphagia, facial congestion, or palpitations. Their deep location and proximity to critical organs make early diagnosis essential [5]. While most retrosternal goitres can be delivered through a cervical incision, those with extensive mediastinal extension, tracheal deviation, or dense adhesions may require sternotomy [2]. This case highlights one such rare presentation necessitating conversion to sternotomy due to deep mediastinal fixation and restricted cervical mobility, underscoring the surgical decision-making challenges in selecting the appropriate approach.

Although numerous reports exist from high-income countries, literature from low- and middle-income settings remains limited, particularly regarding perioperative management in resource-constrained environments. This case therefore contributes to bridging that gap and illustrates context-specific surgical decision-making in a low-resource tertiary setting.

## Case presentation

We present the case of a 55-year-old woman who presented in January 2025 with progressively worsening shortness of breath for more than seven years. She was a known case of hypertension and osteoarthritis. Her dyspnoea had gradually worsened over time, was exacerbated by exertion, and partially relieved by rest. She had undergone thyroid surgery 25 years earlier, although operative records were unavailable. For hypertension, she was taking bisoprolol fumarate 2.5 mg and losartan potassium 25 mg prescribed by her local physician. Her family history was significant for hypertension and diabetes mellitus. Throughout this period, she remained clinically euthyroid, with thyroid function tests confirming normal values. Over the preceding two years, she developed progressive exertional dyspnoea and intermittent dysphagia but denied stridor or voice changes, suggesting slowly progressive compressive symptoms contributing to her delayed presentation.

On physical examination, a small midline neck swelling was noted, becoming more prominent on swallowing. Tracheal deviation to the left was observed, and auscultation revealed reduced air entry in the right upper lung zone. The patient was haemodynamically stable.

Table 1 presents the laboratory results.

**Table 1:** Laboratory results with reference ranges MCH: mean corpuscular haemoglobin, ESR: erythrocyte sedimentation rate, ALT: alanine aminotransferase, AST: alanine aminotransferase, FT3: free triiodothyronine, FT4: free thyroxine, TSH: thyroid-stimulating hormone.

Parameter	Result	Reference range
Haemoglobin (g/dL)	14.0	12.0-16.0
Haematocrit (%)	43.6	36-46
WBC count (×10⁹/L)	8.7	4.0-11.0
MCH (pg)	29	27-33
ESR (mm/hr)	21	<30 (female)
Serum creatinine (mg/dL)	0.9	0.6-1.2
Urea (mg/dL)	33	15-45
ALT (U/L)	36	<40
AST (U/L)	33	<40
Total bilirubin (mg/dL)	0.4	0.2-1.2
Random blood glucose (mg/dL)	122	70-140
FT3 (pmol/L)	5.2	3.1-6.8
FT4 (pmol/L)	14.5	12-22
TSH (mIU/L)	0.42	0.4-4.0
HBsAg	Positive	Negative
Anti-HCV antibody	Positive	Negative
Sputum for AFB	Negative	Negative

**Figure 1:**
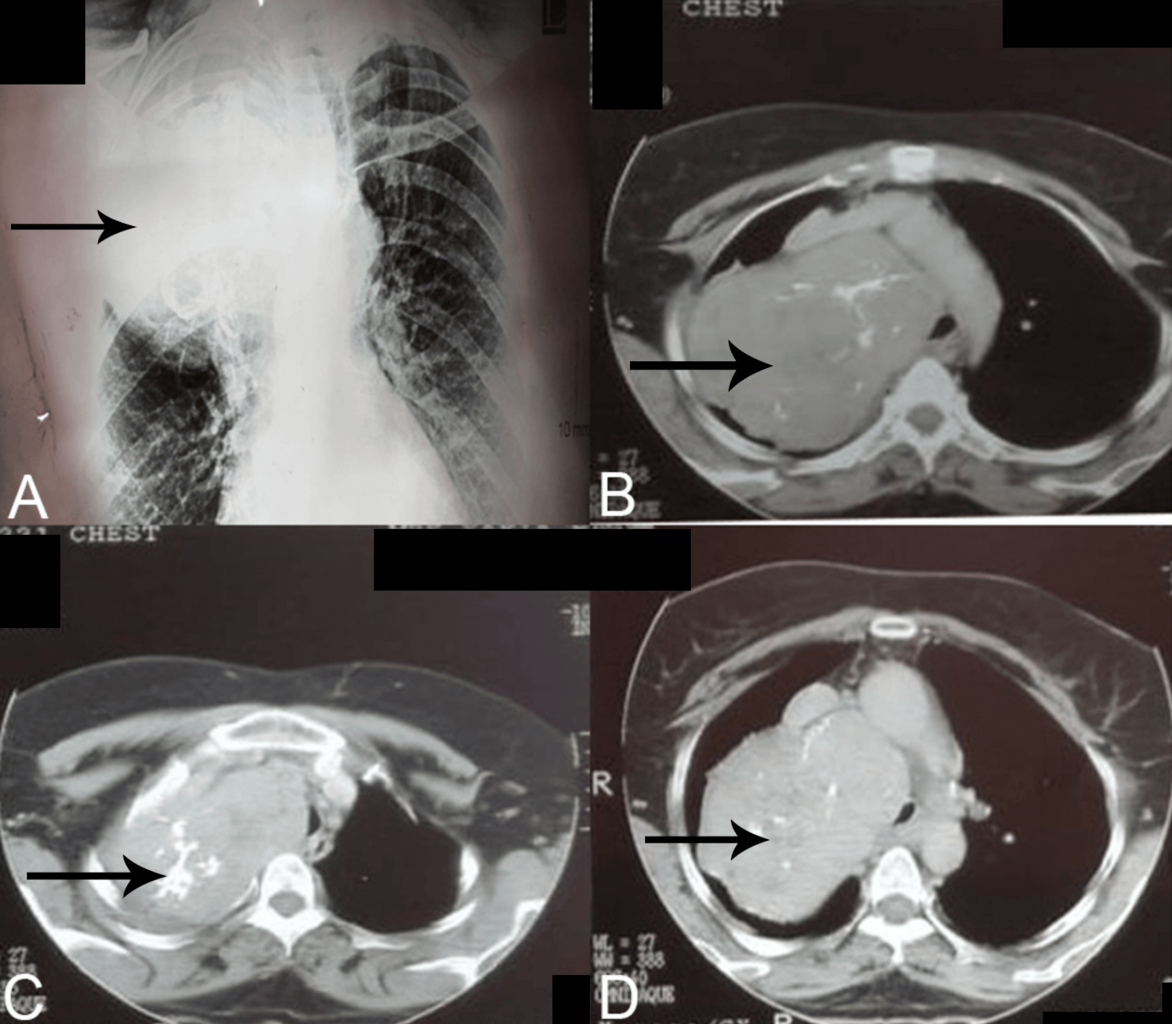
Radiological investigations of the patient. (A) X-ray showing right-sided mass in the region corresponding to the apex of the lung. (B-D) CT scan sections of the patient showing growth in the right side of the thorax.

A chest X-ray revealed a large right-sided intrathoracic mass (Figure 1). Contrast-enhanced CT demonstrated asymmetric enlargement of the thyroid gland, with a right lobe mass measuring 15 × 12 cm extending into the mediastinum and right hemithorax. Multiple coarse calcifications were noted within the mass. The CT also showed approximately 60% tracheal deviation with mild luminal narrowing to an estimated 8 mm at its narrowest point, confirming significant extrinsic compression.

Although cross-sectional imaging strongly suggested a thyroid origin, a Trucut biopsy was performed to exclude mediastinal malignancy and confirm benign pathology before major surgical planning. The procedure was image-guided, conducted with meticulous haemostatic control, and completed uneventfully. Histopathology revealed benign thyroid tissue without lung parenchyma, and immunohistochemistry was positive for PAX-8, confirming thyroid origin.

The patient was subsequently scheduled for surgery. Preoperative optimisation was completed, with clearance obtained from the pulmonology and cardiology teams. Spirometry showed a restrictive pattern (FVC 68% predicted, FEV1/FVC 92%), consistent with extrinsic compression rather than intrinsic pulmonary disease. Given the patient’s dual viral seropositivity (HBsAg and anti-HCV), standard infection control measures and perioperative hepatology input were incorporated. Liver function tests were normal, and viral load was low, permitting safe anaesthesia and surgery with appropriate precautions.

Intraoperative findings

**Figure 2:**
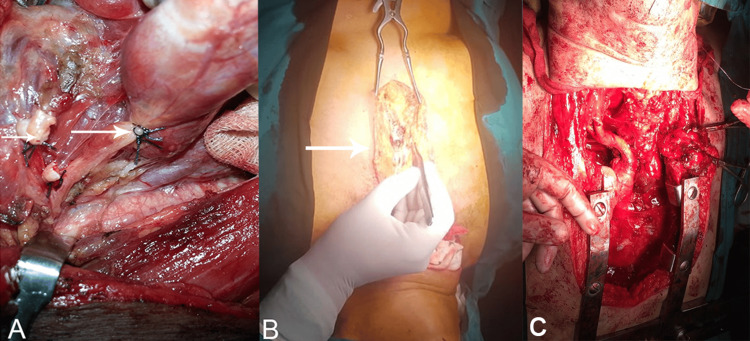
Operative procedure. (A) Surgery was initiated via a cervical approach, with double ligation of the vessels to the gland and mobilisation of the thyroid. (B) Sternotomy was performed to remove the intrathoracic component of the thyroid extending into the posterior mediastinum. (C) Following sternotomy, the gland was completely mobilised and excised, with great care taken to preserve the major vessels.

**Figure 3:**
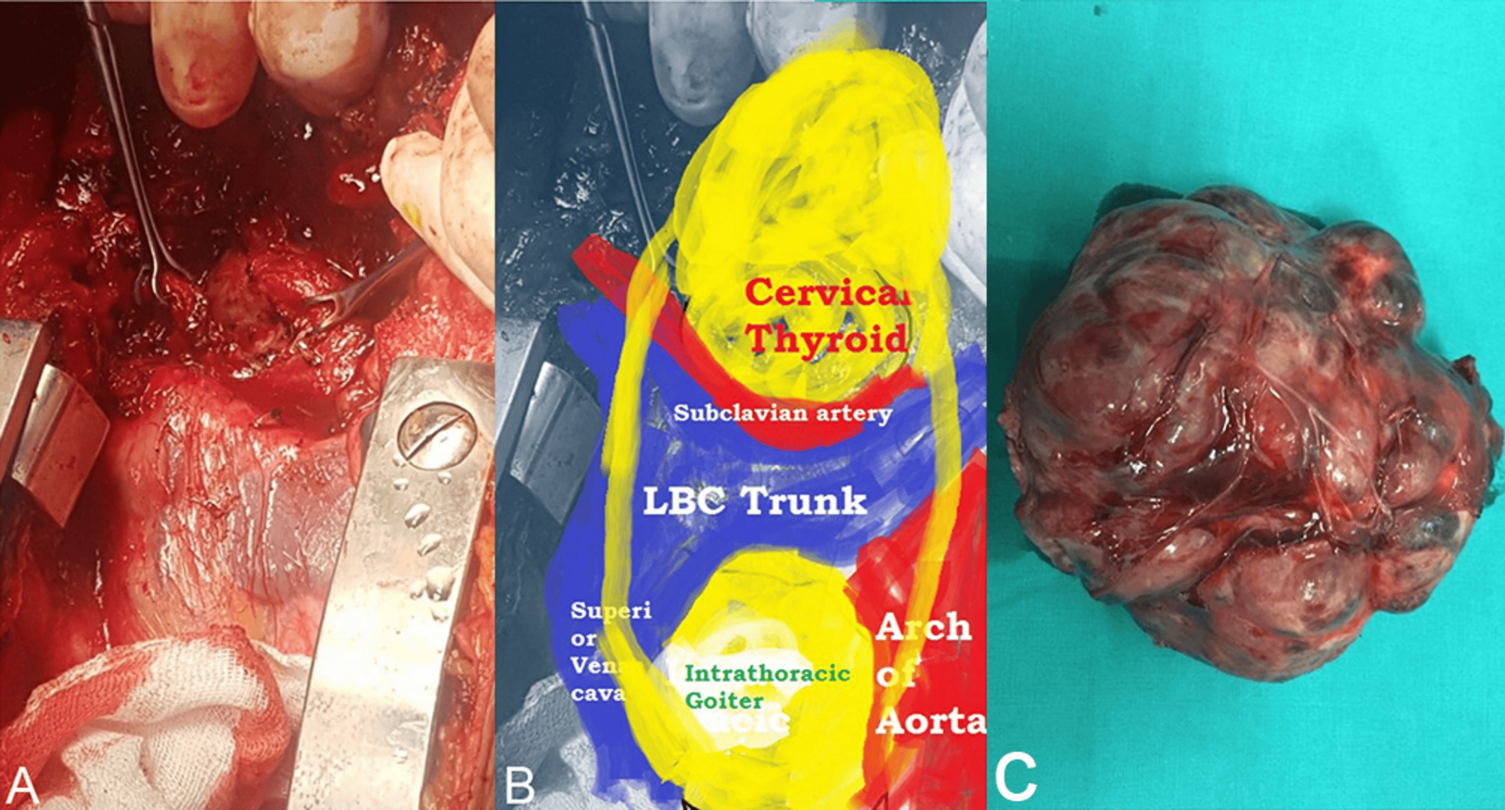
Operative findings. (A) Visualisation of the anatomy after sternotomy, showing thyroid tissue in relation to the major thoracic vessels. (B) Labelled version of A. (C) The intrathoracic thyroid mass removed intact, without fragmentation.

A large mass arising from the right thyroid lobe was found extending into the posterior mediastinum and right thoracic cavity. The mass displaced major mediastinal structures anteriorly and was positioned between the superior vena cava (on the right), the aortic arch (on the left), and anteriorly by the subclavian vessels and left brachiocephalic trunk. Initial dissection through a cervical approach allowed mobilisation of the superior portion of the gland. However, the inferior pole could not be safely mobilised due to dense mediastinal extension and traction-related haemodynamic instability. After multidisciplinary discussion with the anaesthesia and thoracic teams, a decision was made to proceed with partial sternotomy to ensure complete and controlled gland removal. The intrathoracic component was carefully dissected, the brachiocephalic vein was ligated, and meticulous care was taken to preserve surrounding thoracic structures. The mass was fully mobilised and excised. Haemostasis was achieved, and closure was performed in layers. A drain and chest tube were placed (Figures 2 and 3).

Postoperative course

The patient was closely monitored for airway patency, vocal cord function, and calcium levels in the immediate postoperative period. No respiratory compromise or biochemical abnormalities were observed. On postoperative day 2, the patient developed transient atrial fibrillation with a rapid ventricular response. Rate control was achieved with intravenous beta-blocker therapy, and sinus rhythm was restored within 24 hours without recurrence. The chest tube was removed on postoperative day 10. Histopathological examination revealed nodular hyperplasia with colloid-filled follicles of varying sizes, focal haemorrhage, and fibrosis, consistent with multinodular goitre. No evidence of malignancy or capsular or vascular invasion was identified. Post-thyroidectomy hypothyroidism was anticipated, and the patient was started on appropriate levothyroxine replacement therapy with regular endocrine follow-up. The patient has remained asymptomatic on continued follow-up.

## Discussion

The neck is often described as a 'bottomless space', allowing the thyroid gland to enlarge and extend inferiorly into the thoracic cavity. A thyroid mass that descends below the manubriosternal notch is termed a retrosternal or ITG [1]. Part of this mass may remain impalpable, hidden behind the sternum. Regardless of its origin, cervical or intrathoracic, the presence of compressive symptoms such as dyspnoea, dysphagia, or facial congestion is a clear indication for surgical intervention [6]. These symptoms are more frequently observed in ITGs due to their deeper location and limited anatomical space.

Most ITGs can typically be removed through a standard trans-cervical approach. However, in some instances, such as those involving large goitres, posterior mediastinal extension, or recurrent disease, cervical approach alone may carry higher risks, including injury to the recurrent laryngeal nerve, uncontrolled bleeding, and incomplete removal [2]. Posterior mediastinal involvement, as seen here, is even more exceptional and has been rarely described in regional literature. Most retrosternal goitres can be removed via a cervical incision, with only a small proportion (approximately 5%-10%) requiring sternotomy for safe access [3]. Reported complication rates are comparable when the latter is performed in appropriate cases. For these cases, a median sternotomy or combined cervico-thoracic approach is often necessary for safe and effective surgical access [7]. Beyond early diagnosis, the greatest challenge lies in meticulous surgical planning through a coordinated multidisciplinary approach involving general or head and neck surgeons, thoracic surgeons, and anaesthesiologists to ensure safe exposure and airway management.

Posterior mediastinal goitres pose additional challenges due to their proximity to vital thoracic structures [5]. In our case, the mass was closely related to major vessels and necessitated a cervical-sternotomy approach for safe excision. Multidisciplinary teamwork, especially with thoracic surgeons, can be advantageous in managing these complex situations. Sternotomy is also preferred in cases involving suspected malignancy, ectopic goitre, or recurrent disease [7]. Our patient had previously undergone thyroidectomy 25 years ago and developed compressive symptoms approximately 18 years later, which align with the typical timeline seen in recurrent goitre [8]. Recurrence is often attributed to incomplete initial resection or persistent glandular stimulation, particularly in iodine-deficient regions, factors that may also have contributed to this patient's condition.

During surgery, complete removal of the gland in a single intact mass is ideal, as gland fragmentation raises the risk of bleeding and the spread of malignant cells [9]. In our case, the thyroid mass was delivered intact without fragmentation. All vascular pedicles were carefully ligated to reduce the risk of haemorrhage, which remains one of the most significant intraoperative challenges in such cases. Other potential complications include injury to the recurrent laryngeal nerve and parathyroid glands, which may cause vocal cord palsy, hypoparathyroidism, or hypothyroidism. Fortunately, our patient showed no signs of nerve damage or parathyroid dysfunction after surgery. Long-standing ITGs causing significant tracheal compression may also result in tracheomalacia, sometimes necessitating tracheostomy. In our case, there was no evidence of tracheomalacia, which was actively assessed intraoperatively and while extubating, and the airway was managed conservatively.

Seropositivity for hepatitis B and C introduces additional perioperative considerations, including risk of bleeding due to coagulopathy and infection control implications. In our patient, careful pre-operative optimisation and surgical field protection mitigated these risks. Transient atrial fibrillation after major neck or mediastinal surgery is often attributed to intraoperative stress, mediastinal traction, or postoperative catecholamine surges. Early recognition and rate control usually suffice; in this case, no structural cardiac abnormality was found, supporting a stress-related mechanism. The patient remains under annual clinical and ultrasound surveillance to monitor for any residual or recurrent thyroid tissue.

Clinically, the absence of a prominent cervical mass should not exclude the diagnosis of an ITG, particularly in patients presenting with compressive symptoms [10]. As seen in our case, only a minor midline swelling was noted, while imaging revealed a large posterior mediastinal mass. Thoracic imaging plays a crucial role in assessing the extent of disease, evaluating the involvement of adjacent structures, and guiding surgical planning. Early surgical intervention is essential to prevent irreversible complications from prolonged compression. This case adds to the limited regional literature by documenting posterior mediastinal extension necessitating sternotomy in a dual hepatitis-positive patient, a combination rarely described.

## Conclusions

ITGs, especially those extending into the posterior mediastinum, pose a rare yet clinically significant surgical challenge due to their compressive symptoms and proximity to vital mediastinal structures. Our case underscores the importance of maintaining a high index of suspicion in patients with subtle cervical findings but notable respiratory or compressive complaints. Advanced imaging is essential for accurate diagnosis and surgical planning, while a combined cervical and sternotomy approach may be necessary to ensure safe and complete excision. Given the rarity of posterior mediastinal extension, optimal outcomes depend on meticulous preoperative planning through a multidisciplinary team approach and continued long-term follow-up to monitor for recurrence or late complications. Early recognition and prompt surgical intervention remain the cornerstone of preventing long-term morbidity in such patients.
